# Misdiagnosis of a twin pregnancy with double-corner uterine rupture following salpingectomy and protrusion of the amniotic sac as an adnexal cyst: a case report

**DOI:** 10.1186/s12884-020-2773-x

**Published:** 2020-02-03

**Authors:** Jinhua Dong, Yunfei Cao, Qiang Ma, Lili Xue, Weiying Zhu

**Affiliations:** Department of Obstetrics and Gynecology, The Women and Children Hospital Affiliated to Jiaxing University, 2468 Middle Ring Eastern Road, Jiaxing City, 314000 Zhejiang China

**Keywords:** Uterine rupture, Salpingectomy, Intrauterine pregnancy

## Abstract

**Background:**

Salpingectomy-associated uterine rupture during intrauterine pregnancy is rare in the clinic. We report a case of pregnancy with bilateral rupture of the uterine horns after bilateral salpingectomy.

**Case presentation:**

A 30-year-old woman of Han ethnicity presented with right epigastric pain at 28 weeks and 6 days of gestation. Examination by colour Doppler ultrasound showed the following: “Twin live births with normal foetal umbilical artery blood flow indexes and a 183 mm × 112 mm anechoic zone in the right front of the uterus”. Initially, we made an incorrect judgement wherein we considered the amniotic sac that was protruding into the abdominal cavity to be an adnexal cyst. Fortunately, the diagnosis of uterine rupture was confirmed before the protruded amniotic sac broke. The mother did not bleed much, and the twin foetuses survived in our case.

**Conclusion:**

A previous history of salpingectomy via laparoscopy could be a risk factor for uterine rupture in pregnant women. Attention should be paid to rare complications of pregnancy. To avoid adverse events, we should pay special attention to women with a history of laparoscopic salpingectomy who complain about abdominal discomfort and offer them a relevant ultrasound examination.

## Background

Usually, patients with a previous history of uterine surgery (including caesarean section or hysteromyomectomy) have a greater risk of rupture, while those without scars have a very low risk of rupture [1, 2]. The typical uterine rupture is often accompanied by severe abdominal pain, vaginal bleeding, loss of the foetal heartbeat, and uterine diminution. Generally, when the aforementioned symptoms occur, the foetus dies, and the mother’s life is threatened [3]. Uterine rupture during intrauterine pregnancy due to salpingectomy is a rare and unusual type of rupture, and in most cases, the foetus dies before detection. In the case described here, we were lucky to have the opportunity to confirm the diagnosis and avoid the disastrous consequences of the condition.

## Case presentation

**Fig. 1:**
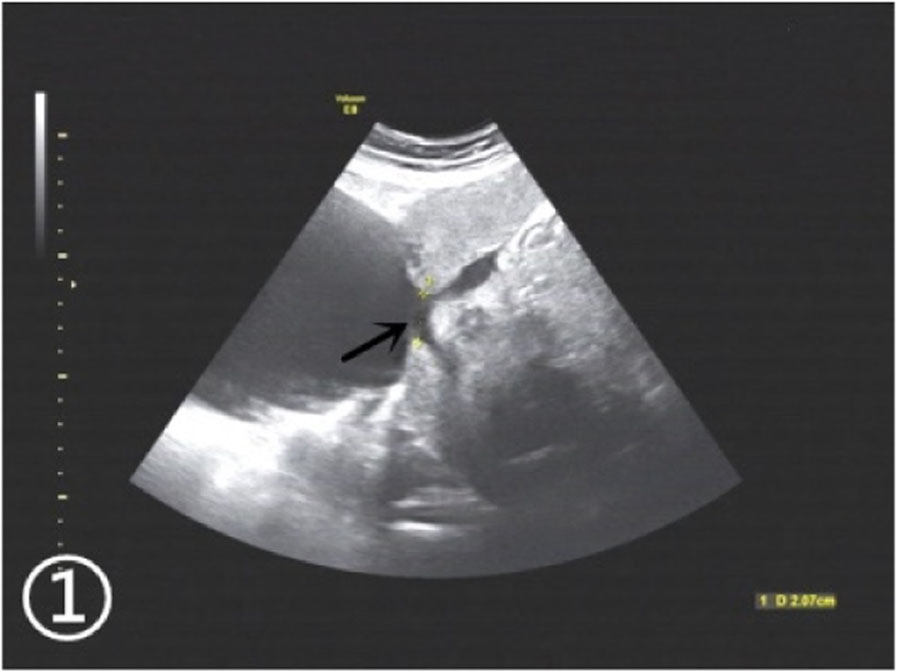
Ultrasound scan image: myometrial defect at the right uterine horn and hypoechoic liquid dark area connected to the uterine cavity

**Fig. 2:**
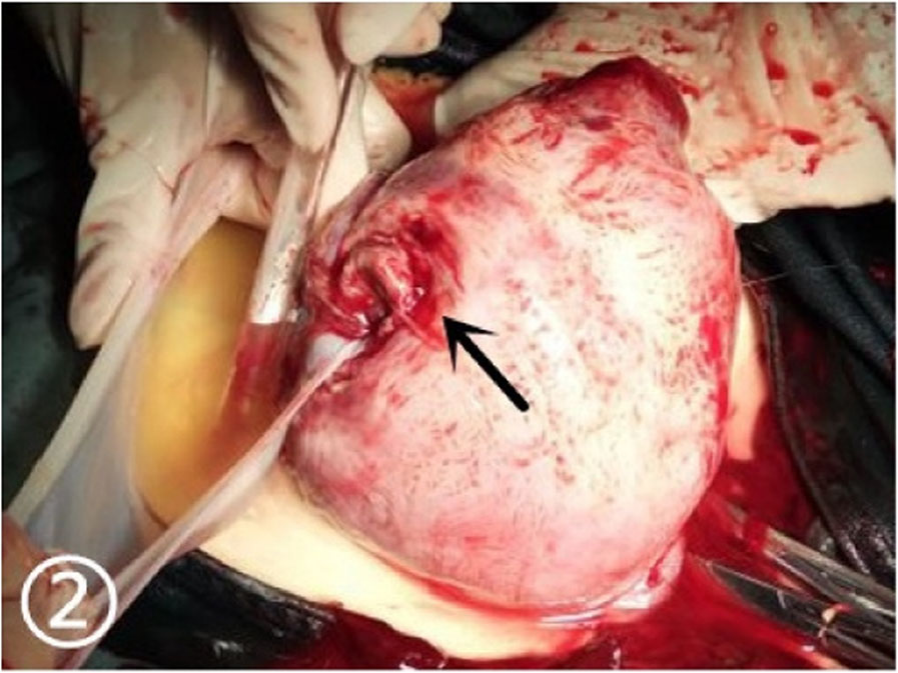
Intraoperative findings: Ruptured right uterine horn; amniotic membrane and amniotic fluid

**Fig. 3:**
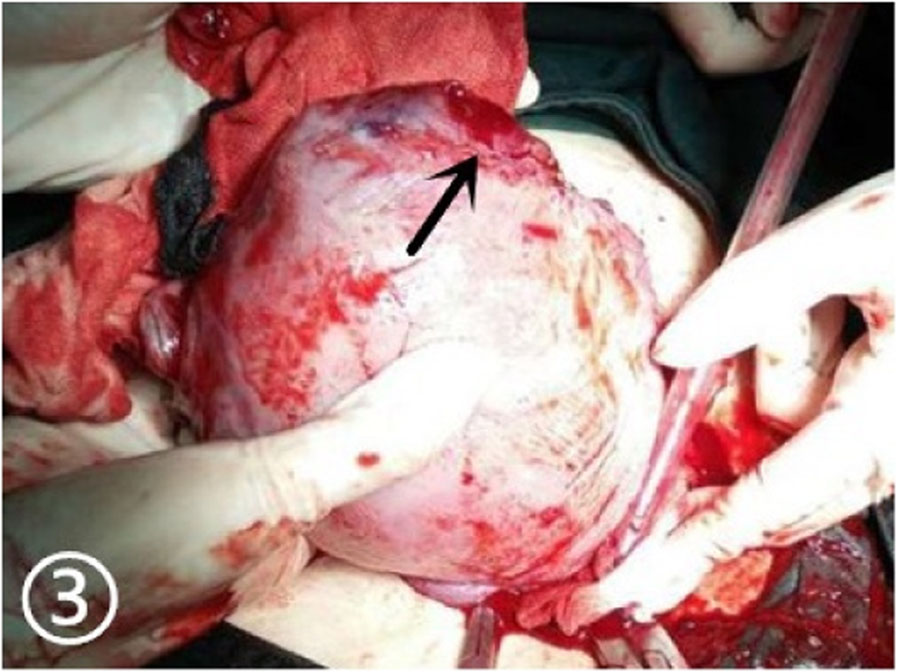
A 0.5 cm × 0.5 cm rupture in the left corner of the uterus with a small amount of active haemorrhage. The black arrow indicates the rupture site

A 30-year-old woman of Han ethnicity, at 28 weeks and 6 days of gestation, complained of right epigastric pain 3 h before being seen and was immediately admitted to the hospital on May 13, 2016. She had not given birth but had a history of two right fallopian tube pregnancies. Fearing that she would have the same experience again, she gave up trying for a natural pregnancy and underwent laparoscopic bilateral salpingectomy using bipolar electrosurgical coagulation in a local hospital. The operation was a simple salpingectomy, without the involvement of the uterine horn. Therefore, she received a recommendation for in vitro fertilization and embryo transfer after four months. Two frozen embryos were transplanted, and colour Doppler ultrasonography showed “double chorionic double amniotic sac twins” at 6 weeks of gestation. Until May 14, there had been no special abnormalities during her prenatal examinations. On the day of admission, there was no obvious cause of her right upper abdominal minimal and obscure pain. The pain lasted for 20 min, after which she experienced relief without lower abdominal pain. The patient had no nausea or vomiting, no diarrhoea, and no symptoms of vaginal bleeding or fluid leakage. The foetal movement was normal. Physical examination revealed the following: P, 123 beats/min; R, 19 beats/min; BP, 113/68 mmHg; and T: 36.4 °C. No significant abnormalities in cardiac and pulmonary auscultation were found. No tenderness or rebound pain was observed in the abdomen. Colour Doppler ultrasound showed “Twin live births with normal foetal umbilical artery blood flow indexes and a 183 mm × 112 mm anechoic zone in the right front of the uterus”. We considered the cystic mass on the right anterior side of the uterus to be an adnexal cyst. Routine blood tests on the day after admission showed the following: WBC, 18.7*10^9/L; NE%, 84.8; and HGB, 104 g/l. On May 15, the patient felt fewer foetal movements than before without any other discomfort. We re-examined the baby by colour Doppler ultrasound. Two live foetuses were found in the uterus. The muscular layer of the right uterine wall was ruptured with a width of approximately 21 mm. On the right side of the uterus, there was a 139 mm × 118 mm anechoic area, which was connected to the uterus. In the anechoic area, foetal limbs and strip-shaped strong echoes were observed (Fig. 1). There was no amniotic fluid around the twins. At that time, no obvious effusion was found in the abdominal cavity of the pregnant woman. Emergency caesarean section was performed under combined spinal-epidural anaesthesia. Two male infants weighing 1260/1240 g were delivered through a lower uterine incision. The Apgar scores at 1 min and 5 min were 6/7 and 6/6, respectively. Intraoperative examination showed that the amniotic sac, with a volume of approximately 13 cm × 10 cm × 10 cm, was bulging from the right corner of the uterus to the abdominal cavity, with clear amniotic fluid (Fig. 2). At the same time, we found a 0.5 cm × 0.5 cm rupture in the left corner of the uterus with a small amount of active haemorrhage (Fig. 3). We repaired and sutured the uterine rupture on both sides and increased the anti-inflammatory treatment after the operation. The patient recovered well and was discharged from the hospital on the 7th day after surgery.

## Discussion and conclusion

Uterine rupture during intrauterine pregnancy after salpingectomy is a rare clinical adverse event [4]. To our knowledge, no more than 10 cases have been reported in the literature, most of which are unilateral ruptures of a uterine horn [5]. Only one case of spontaneous rupture of both corners of the uterus after bilateral salpingectomy via laparoscopy was reported, by Inovay and colleagues [6]. The rupture occurred 14 weeks after in vitro fertilization, with vaginal spotting and intense abdominal pain; unfortunately, when a defect in the uterine wall was suspected, the foetus had died. In our case, there was bilateral rupture of the uterine horns, the amniotic sac did not break when it protruded from the ruptured uterus, and the twin foetuses survived. This type of case is extremely rare in clinical practice.

Uterine rupture in patients without apparent risk factors is associated with non-specific signs and symptoms that can postpone the diagnosis. Atypical uterine rupture cannot be detected in time, and the delay is often the main cause of the adverse outcomes of mothers and infants [7]. In our case, as the procedure was only a simple bilateral salpingectomy and did not involve the corners of the uterus, we neglected the possibility of uterine rupture. The symptom of abdominal pain was obscure and disappeared quickly, which confounded the diagnosis. While evaluating previous cases of uterine rupture following salpingectomy, we found that a few of them had no obvious symptoms and that their ruptures were only unintentionally found during caesarean section [5]. Sometimes, the abdominal pain is similar to that of a threatened preterm delivery, so the possibility of uterine rupture is easy to ignore. When the condition progresses, it is often too late to alleviate the shock symptoms. In our case, rupture of the uterus was found on the second ultrasound examination; however, the patient had no discomfort but did show decreased foetal movement. Surprisingly, the amniotic sac protruded from the rupture of the uterus for 2 days without rupturing, until there was no amniotic fluid around the foetus, resulting in fewer foetal movements. Although this complication is rare, it can be detected and diagnosed by ultrasound in early pregnancy, even in asymptomatic patients, as long as there is sufficient diagnostic awareness.

Why does uterine rupture occur after salpingectomy? In addition to the surgical method, the time interval between salpingectomy and conception is very important. It is generally believed that laparoscopic salpingectomy can cause thermal damage, which results in defects in the corners of the uterus [8]. The recommended pregnancy interval is usually 2 years after uterine injury [9]. The interval has been reported to be less than 12 months for 67% of cases of uterine rupture in a non-ectopic pregnancy group (after salpingectomy) [5]. The cause of uterine rupture in this case was considered to be pregnancy at only 4 months after laparoscopic bilateral salpingectomy. Perhaps, poor healing after salpingectomy and rapid uterine enlargement in the twin pregnancy resulted in rupture of the injury sites.

By studying this case, we have gained some experience and learned several lessons. The uterine rupture had occurred by the time of the first ultrasound examination but was not clearly diagnosed by ultrasound. If the patient’s condition progressed rapidly, the consequences would be worrying. Therefore, when performing ultrasound scans, the cystic mass, especially its relationship to the uterus and its blood flow, should be carefully evaluated. If necessary, an experienced ultrasound doctor should be consulted. In addition, rupture of the left uterine horn was not diagnosed by the preoperative ultrasound in this case. The rupture of the right uterine horn was confirmed, and the ultrasound doctor was eager to complete the ultrasound examination without careful resolution. In addition, this may be because the rupture port was too small to be examined by ultrasound.

A previous history of salpingectomy via laparoscopy could be a risk factor for uterine rupture in pregnant women. To avoid adverse events, we should pay special attention to women with a history of laparoscopic salpingectomy who complain of abdominal discomfort and offer them a relevant ultrasound examination.

## Data Availability

The datasets created and/or analysed during the current study are available from the corresponding author on reasonable request.

## Abbreviations

HGB: Haemoglobin
NE%: Percentage of neutrophils
WBC: White blood cell
